# MicroRNA 10a Marks Regulatory T Cells

**DOI:** 10.1371/journal.pone.0036684

**Published:** 2012-05-18

**Authors:** Lukas T. Jeker, Xuyu Zhou, Kseniya Gershberg, Dimitri de Kouchkovsky, Malika M. Morar, Gustavo Stadthagen, Anders H. Lund, Jeffrey A. Bluestone

**Affiliations:** 1 Diabetes Center and the Department of Medicine, University of California San Francisco, San Francisco, California, United States of America; 2 Biotech Research and Innovation Centre, University of Copenhagen, Copenhagen, Denmark; Université Paris Descartes, France

## Abstract

MicroRNAs (miRNAs) are crucial for regulatory T cell (Treg) stability and function. We report that microRNA-10a (miR-10a) is expressed in Tregs but not in other T cells including individual thymocyte subsets. Expression profiling in inbred mouse strains demonstrated that non-obese diabetic (NOD) mice with a genetic susceptibility for autoimmune diabetes have lower Treg-specific miR-10a expression than C57BL/6J autoimmune resistant mice. Inhibition of miR-10a expression *in vitro* leads to reduced FoxP3 expression levels and miR-10a expression is lower in unstable “exFoxP3” T cells. Unstable *in vitro* TGF-ß-induced, iTregs do not express miR-10a unless cultured in the presence of retinoic acid (RA) which has been associated with increased stability of iTreg, suggesting that miR-10a might play a role in stabilizing Treg. However, genetic ablation of miR-10a neither affected the number and phenotype of natural Treg nor the capacity of conventional T cells to induce FoxP3 in response to TGFβ, RA, or a combination of the two. Thus, miR-10a is selectively expressed in Treg but inhibition by antagomiRs or genetic ablation resulted in discordant effects on FoxP3.

## Introduction

The successful use of regulatory T cells (Tregs) in various preclinical models of inflammatory and autoimmune disease has led to clinical trials using Tregs to treat or prevent graft versus host disease, type 1 diabetes (T1D) and transplant rejection [1]. However, recent studies have suggested that mice and humans harbor a small population of Tregs that lose the expression of the crucial transcription factor, FoxP3, leading to instability, especially under certain inflammatory conditions [2]. The so-called exFoxP3 cells have been shown to be potentially pathogenic [2]. The basis for this instability and its effects on Treg suppressive activity remains unclear.

microRNAs (miRNAs) are short, single-stranded RNA molecules involved in maintaining immune homeostasis, particularly during stress, such as inflammation, by fine-tuning gene expression post-transcriptionally [3]. Dicer-deficient Tregs are extremely unstable, unable to maintain immune homeostasis leading to a scurfy-like, autoimmune disease [4], [5], [6]. In addition to Dicer, Drosha [6] and DGCR8 (Jeker and Bluestone, unpublished observation), two proteins of the microprocessor complex involved in the biogenesis of miRNAs, are also essential for Treg function. Thus, as a class of regulatory genes, miRNAs are critical for Treg development, function and lineage stability [4], [5], [6], [7]. In contrast, very little is known about the role of individual miRNAs in Tregs.

## Results

### Treg miRNA Expression Signature

**Figure 1:**
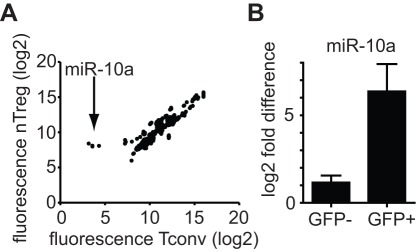
Treg miRNA expression signature. a) miRNA microarray analysis of CD4^+^CD25^-^GFP^-^ (Tconv) and CD4^+^CD25^hi^GFP^+^ (Treg cells) purified from lymph nodes from female FoxP3-GFP-hCre reporter mice. Shown are 4 technical replicates from the same slide (one biologic replicate). b) qPCR of relative miR-10a expression by sorted Tconv (GFP^-^) and Treg (GFP^+^). One representative example of >7 independent experiments from >7 independent biologic replicates. Error bars: SD of technical triplicates.

Only a limited number of miRNAs have been previously reported to be differentially expressed in Treg versus Tconv [7]. We generated a Treg-specific miRNA profile by using FoxP3-driven GFP reporter mice [4] to distinguish Treg from Tconv cells. miRNA microarray analysis of Tconv (>99% purity) and Treg cells (>98% purity) identified mostly miRNAs expressed in both cell types with some being expressed at quantitatively different levels (Fig. 1a and Table S1). miR-10a was the only miRNA exclusively detected in Treg when compared to Tconv (Fig. 1a). The differential expression of miR-10a was confirmed by qPCR on RNA from freshly purified CD4^+^GFP^-^ Tconv and CD4^+^GFP^+^ Treg populations from FoxP3-GFP reporter [8] and FoxP3-GFP-hCre reporter mice (Fig. 1b). miR-10a was readily detected in Treg cells (n>7 independent experiments). In contrast, there was either no or minimal signal with high variability when RNA from Tconv cells was examined. Similar results were observed with Tregs isolated from non-transgenic C57BL/6J (B6) mice isolated based on phenotypic expression markers (CD4^+^CD25^+^CD62L^hi^ cells) (data not shown). miR-10a expression was similar in Tregs independent of sex or age (5 weeks to 16 weeks old mice) (data not shown).

### miR-10a Marks Treg Cells

**Figure 2:**
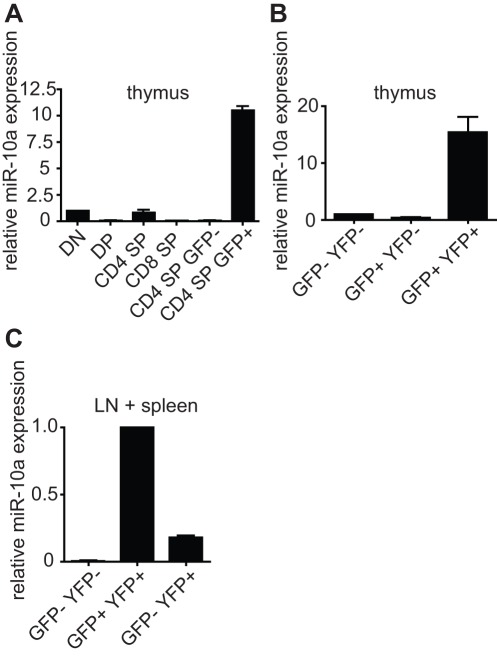
miR-10a marks Treg cells. qPCR analysis of relative expression of miR-10a in purified T cells. a) Thymocytes: CD4^-^CD8^-^ double negative (DN), CD4^+^CD8^+^ double positive (DP), CD4^+^CD8^-^ single positive (CD4SP), CD8^+^CD4^-^ single positive (CD8SP), CD4^+^CD8^-^FoxP3-GFP^-^ (CD4SP GFP^-^) and CD4^+^CD8^-^FoxP3-GFP^+^ (CD4 SP GFP^+^). b) CD4 SP FoxP3-GFP^-^R26YFP^-^ (GFP^-^YFP^-^), CD4 SP FoxP3-GFP^+^R26YFP^-^ (GFP^+^YFP^-^) and CD4 SP FoxP3-GFP^+^R26YFP^+^ (GFP^+^YFP^+^) thymocytes. c) CD4^+^GFP^-^YFP^-^ (Tconv), CD4^+^GFP^+^YFP^+^ (nTreg) and CD4^+^GFP^-^YFP^+^ (exFoxP3) cells purified from pooled LN and spleen. Shown is one representative experiment from four (a) and two (b, c) independent experiments. Error bars: SD of triplicates.

To validate the expression profiling, we analyzed miR-10a expression by qPCR in various purified cell populations from FoxP3-GFP or FoxP3-GFP-hCre reporter mice. We purified CD4^-^CD8^-^ double negative (DN), CD4^+^CD8^+^ double positive (DP), CD4^+^CD8^-^ single positive (CD4 SP) or CD4^-^CD8^+^ single positive (CD8 SP) thymocytes by FACS (data not shown). Little or no miR-10a signal was observed in DN thymocytes and no signal was detected in DP or CD8 SP thymocytes. CD4 SP cells expressed miR-10a levels comparable to DN cells (Fig. 2a). However, when the CD4^+^ SP cells were subdivided into GFP^-^ and GFP^+^, i.e. FoxP3 expressing cells versus Tconv thymocytes, a clear signal was observed only in the CD4^+^ SP GFP^+^ natural Treg (nTreg) population (Fig. 2a). Reproducibility of this experiment is shown in Fig. S1. Next we examined the relationship between miR-10a and FoxP3 expression. We took advantage of the recently described lineage tracing strategy [2]. Individual CD4^+^ thymocyte subpopulations were purified including GFP^-^YFP^-^ cells that do not express FoxP3 (and never did), GFP^+^YFP^-^ cells where FoxP3 was turned on but cre activity was not sufficient to result in detectable YFP protein levels yet, and GFP^+^YFP^+^ which represented actively expressing “bona fide” nTreg as a means to determine the temporal expression of miR-10a during thymic nTreg development (Fig. 2b). Neither GFP^-^YFP^-^ nor GFP^+^YFP^-^ cells expressed miR-10a, while there was significant miR-10a expression in thymic GFP^+^YFP^+^ Treg suggesting that miR-10a expression occurs temporally after FoxP3 expression (Fig. 2b). We can not exclude that a small part of the miR-10a signal detected in GFP^+^YFP^+^ Treg stems from recirculating Treg. Nevertheless, the result demonstrates that within thymocytes miR-10a expression is restricted to Treg (independent of their origin of generation) and FoxP3 expression precedes miR-10a expression.

Next, we analyzed miR-10a expression in peripheral T cell subsets. As noted previously, miR-10a is expressed in Treg but not bulk CD4^+^ or CD8^+^ Tconv cells (data not shown). Of note, miR-10a levels in exFoxP3 cells (GFP^-^YFP^+^) were equal or slightly higher to Tconv (GFP^-^YFP^-^) levels but much lower than in nTreg cells (GFP^+^YFP^+^) (Fig. 2c) illustrating a tight correlation between FoxP3 and miR-10a expression suggesting that miR-10a may be a stabilizing factor of Foxp3 expression. Reproducibility of experiments shown in Fig. 2b and Fig. 2c is shown in Fig. S2. In summary, miR-10a marks Treg cells in thymocytes, LN and spleen but not other T cell subsets such as early thymocytes, bulk CD4^+^, and CD8^+^ T cells.

### All-trans Retinoic Acid but Not TGF-ß Induces miR-10a in CD4^+^ T Cells

**Figure 3:**
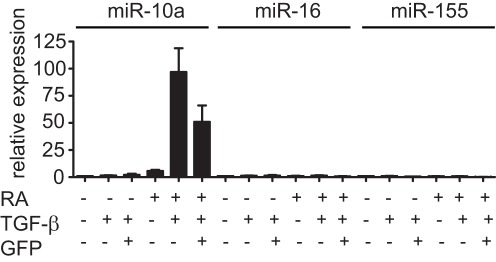
All-trans retinoic acid but not TGF-ß induces miR-10a in CD4^+^ T cells. FACS-purified CD4^+^CD62L^hi^GFP^-^ cells from FoxP3-GFP reporter mice were cultured with plate-bound anti-CD3 and anti-CD28 antibodies +/− TGF-ß and/or retinoic acid. After 72 h the CD4^+^GFP^-^ and CD4^+^GFP^+^ cells were purified by flow cytometry for RNA extraction. miRNA levels were assessed by qPCR in technical triplicates. Shown is a representative experiment of two independent experiments. Error bars: SD of triplicates.

The highly specific miR-10a expression in Tregs led us to investigate inductive signals. Neither T cell activation by stimulating purified CD4^+^CD25^-^ cells with anti-CD3 and anti-CD28 did induce miR-10a (data not shown) nor *in vitro* skewing into Th1 and Th17 cells increased miR-10a expression above levels seen under neutral culture conditions (data not shown) suggesting that miR-10a expression is limited to the FoxP3^+^ T cell lineage. Multiple studies have shown that *in vitro* T cell activation in the presence of TGF-ß can induce FoxP3 in Tconv to generate CD4^+^FoxP3^+^ iTreg. However, the transcriptome of iTreg significantly differs from nTreg indicating that additional signals might be involved during physiological nTreg development [9]. Importantly, iTreg are not as stable as nTreg since withdrawal of TGF-ß results in a loss of FoxP3 while ex vivo purified nTreg maintain high FoxP3 expression independent of TGF-ß [10]. Since we detected miR-10a in nTreg but reduced levels in unstable exFoxP3 cells (Fig. 2a-c) we tested miR-10a levels in TGF-ß-induced iTreg cells, hypothesizing that like exFoxP3 cells they would have reduced miR-10a expression. Indeed, despite substantial FoxP3 (GFP) induction (data not shown) miR-10a was not induced in GFP^+^ cells (Fig. 3). These results suggest that FoxP3 expression alone is not sufficient to drive miR-10a expression.

All-trans-retinoic acid (RA), the oxidized form of Vitamin A has been shown to play a role in the conversion of Tconv into FoxP3^+^ adaptive regulatory T cells (aTreg) and may play a critical role in iTreg stability [11]. Although the precise molecular mechanism remains controversial, it is well accepted that the net result of combined RA/TGF-ß-treatment of Tconv is enhanced FoxP3 induction compared to TGF-ß alone. We tested if RA could induce miR-10a in T cells. RA alone or in combination with TGF-ß induced miR-10a (Fig. 3). Although RA-treated GFP^-^ (FoxP3^-^) cells did express low levels of miR-10a in the absence of TGF-ß, the presence of TGF-ß synergized to maximally induce miR-10a. This effect was specific as expression of miR-16 and miR-155 was not induced (Fig. 3). Reproducibility of this experiment is shown in Fig. S3. In summary, RA but not TGF-ß alone can induce miR-10a in conventional CD4^+^ T cells.

### Treg-specific miR-10a Modulates FoxP3 Stability *in vitro*

**Figure 4:**
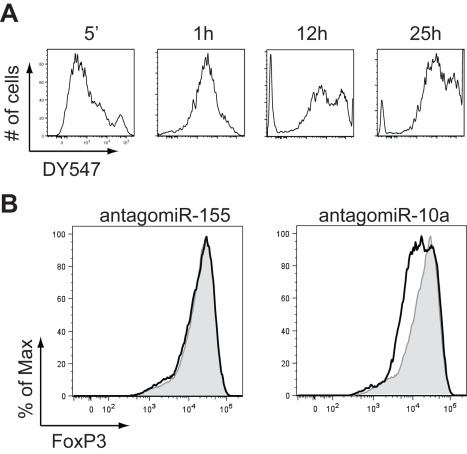
Treg-specific miR-10a modulates FoxP3 stability *in vitro*. FACS-purified CD4^+^CD62L^hi^GFP^+^ cells from FoxP3-GFP reporter mice were cultured with anti-CD3 and anti-CD28 beads and 2000U IL-2/ml for 48 h. Time course of antagomiR-DY547 transfection in cultured Treg (a). FoxP3 staining 48 h after culture in the presence of 50 µg/ml antagomiR-155 or antagomiR-10a (b). Grey shaded histograms represent nontransfected cells, black lines represent cells treated with antagomiR. Shown is one experiment (a) and one representative experiment from three (b) independent experiments.

Our understanding of the molecular mechanisms that maintain Treg stability remain uncertain although miRNAs do stabilize Treg lineage identity [4]. Based on its expression, we hypothesized that miR-10a might facilitate/maintain high and stable FoxP3 levels. Therefore, we cultured nTreg in the presence of antagomiR-10a or antagomiR-155 [12]. Antagomirs specifically inhibited the targeted miRNA (data not shown). Fluorescently labeled (DY547) antagomiR-10a demonstrated a very high transfection rate (>90%) as previously reported for other cell types (Fig. 4a) [13]. Uptake was evident within a few minutes after treatment and neared saturation after 12 h (Fig. 4a). AntagomiR-10a treatment of nTreg led to a dose-dependent reduction of GFP (data not shown) and FoxP3 in nTregs compared to antagomiR-155 (Fig. 4b). CD4 expression was not changed by any antagomiR confirming the specificity of the inhibitors (data not shown). Together these results suggest that high FoxP3 levels in nTreg cells are positively controlled by miR-10a.

### miR-10a is Dispensable for TGFβ and Retinoic Acid-mediated FoxP3 Induction

**Figure 5:**
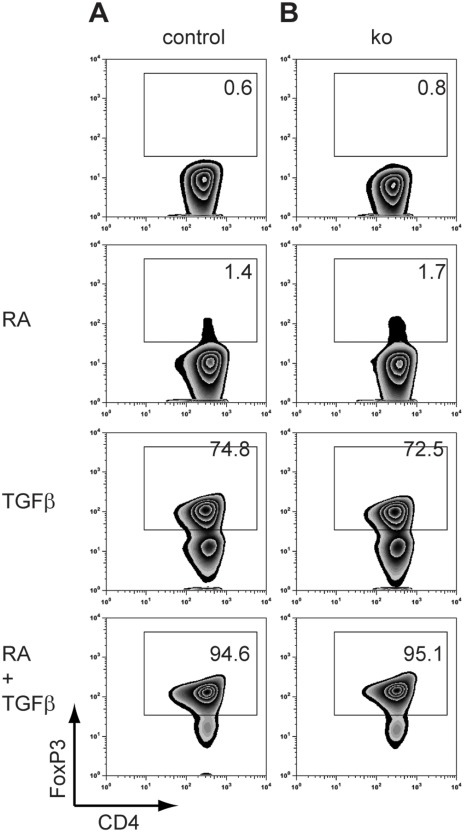
miR-10a is dispensable for TGFβ and retinoic acid-mediated FoxP3 induction. Naïve CD4^+^CD25^-^CD62L^hi^ Tconv were activated with anti-CD3 and anti-CD28 in the presence of RA, TGFβ or a combination of the two. Cells were from wildtype (“control”) or littermate miR-10a-deficient (“ko”) mice. Representative of 4 independent experiments.

To test if miR-10a was required for RA-mediated iTreg induction we purified naïve CD4^+^CD62L^hi^25^−^ Tconv and activated them in vitro with anti-CD3 and anti-CD28 mAb in the presence of RA, TGFβ or a combination of the two. RA induced FoxP3 in very few cells. TGFβ induced FoxP3 in >70% of cells and combining RA and TGFβ induced FoxP3 in almost all cells (Fig. 5a). However, there was no difference between miR-10a sufficient and miR-10a-deficient cells (Fig. 5b). Thus, miR-10a is dispensable for FoxP3 induction.

### miR-10a Expression in Treg Inversely Correlates with Susceptibility to Autoimmune Disease

**Figure 6:**
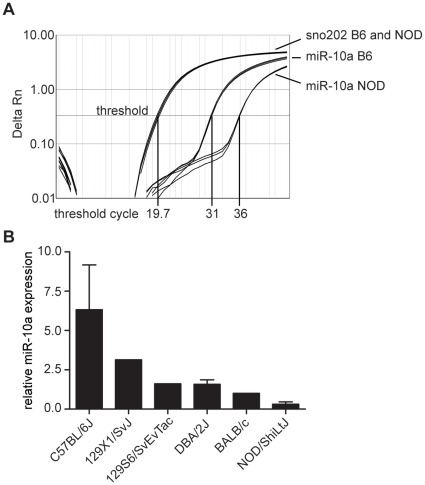
miR-10a expression in Treg inversely correlates with susceptibility to autoimmune disease. qRT-PCR for miR-10a expression by FACS-purified CD4^+^CD25^+^CD62L^hi^ Treg. a) Amplification plots for miR-10a on Treg cDNA from B6 and NOD mice. The sno202 signal for B6 and NOD completely overlapped. The signal for Tconv is comparable to miR-10a in NOD Treg (data not shown). b) Relative miR-10a expression in Treg from B6, 129X1/SvJ, 129S6/SvEvTac, DBA/2J, BALB/c and NOD/ShiLtJ mice. Bars represent means of pooled data from 3 (B6), one (129X1/SvJ), one (129S6/SvEvTac), 2 (DBA/2J), one (BALB/c) and 3 (NOD/ShiLtJ) biologic replicates. Error bars: SEM. All samples were normalized to miR-10a expression in BALB/c mice.

NOD mice have increased frequencies of exFoxP3 cells in islets, an indication of a defect in Treg stability [2]. Therefore, we determined miR-10a expression levels in diabetes-prone NOD and autoimmune-resistant B6 Treg cells (CD4^+^CD25^+^CD62L^hi^). As observed in non-autoimmune prone mice (Fig. 1 and 2), NOD Treg expressed miR-10a but at much lower levels than their B6 counterparts (Fig. 6a). To test whether miR-10a levels in Treg cells correlated with autoimmune susceptibility, we screened various inbred mouse strains for Treg-specific miR-10a expression. Treg from autoimmune-resistant B6 mice had the highest miR-10a expression while Treg from autoimmune-prone NOD mice had the lowest (Fig 6b). Mouse strains with more variable susceptibility to autoimmune disease (BALB/c, 129, DBA/2J) had intermediate expression levels. Thus, we found an inverse correlation of Treg-specific miR-10a expression with susceptibility to autoimmune disease.

## Discussion

Given the opposing roles of regulatory versus conventional helper CD4^+^ T cells, it is puzzling how few protein-coding genes are differentially expressed among these T cell subsets. Most differences are quantitative rather than qualitative [9], yet, the sum of differential gene expression is sufficient to imprint highly specialized functions in each subset. Likewise, the only miRNA specific for Treg cells was miR-10a. Interestingly, miR-10a expression seems to be specifically induced in Treg since the two flanking protein-coding genes are not detectable (HoxB5) or barely expressed (HoxB4) in naive, Th1, Th2, Th17, induced Treg (iTreg) or nTreg [14]. These observations suggest that thymic Treg sense a signal to actively and specifically induce miR-10a.

The expression data suggests a correlation with more stable Treg and combined with the *in vitro* data supports the possibility that this miRNA may be involved in long-term maintenance of Treg stability. Interestingly RA, secreted by a specialized subset of CD103^+^ dendritic cells, is important for inducing Treg in the gut [11], [15], [16]. More importantly, in contrast to TGF-ß alone, the combination of TGF-ß and RA not only increases the frequency of FoxP3 expressing T cells *in vitro* but the induced FoxP3^+^ cells are more stable, particularly in an inflammatory environment [11], [17]. Given the strong induction of miR-10a by RA, the added benefit of RA might partially be accomplished through miR-10a, which is embedded in the HoxB cluster, a genomic region with known RA responsiveness. In a pancreatic cancer cell line RA directly binds to a retinoic acid response element (RARE) in the HoxB cluster and induces miR-10a [18]. Therefore, it seems likely that in T cells RA directly induces miR-10a through direct binding of RARE elements in the HoxB/miR-10a locus. Pharmacologic miR-10a inhibition did indeed inhibit the expression level of miR-10a. However, miR-10a-deficient mice are fertile and age without obvious phenotypic abnormalities (Stadthagen & Lund, unpublished results). Treg numbers and phenotype appear normal under homeostatic conditions (data not shown) and miR-10a was dispensable for FoxP3 induction in naïve T cells. Furthermore, miR-10a-deficient Treg cells suppress colitis in an adoptive transfer model (Jeker & Bluestone, unpublished results). Yet, the importance of this miRNA may only be apparent under certain pathogenic or other stress settings [3], [19], [20] and deKouchkovsky et al., MS submitted. This notion is supported by the fact that NOD Treg express very low levels of miR-10a without NOD mice developing scurfy-like disease and miR-10a-deficient Treg are present in normal numbers and express normal levels of FoxP3 (data not shown).

How can the discrepancy between in vitro inhibition of miR-10a expression with antagomiRs versus genetic ablation be explained? It is possible that antagomiR-10a had off-target effects, e.g. inhibition of miR-10b and miR-125a, which has sequence homology or interaction with other targets. In fact, antagomiR-125a did reduce FoxP3 expression to some extent (although to a lesser degree than antagomiR-10a) but the combination of antagomiR-10a and antagomiR-125a did not further enhance FoxP3 downregulation (data not shown). Alternatively, miR-10b might compensate for the absence of miR-10a in miR-10a-deficient Treg, since miRNAs are often redundant [21]. Temporally controlled conditional miR-10a ablation might reveal its function [22]. Finally, miR-10a might be expressed and functional only in a subset of Treg as it has become clear that CD4^+^CD25^+^FoxP3^+^ cells are heterogeneous and may be functionally diverse, depending on tissue location and activation status [23], [24].

In summary, further studies are required to investigate miR-10a function *in vivo*. Despite an intriguing expression pattern, we were not able to define the miR-10a function in Treg in vivo. Importantly, our results suggest that phenotypic effects obtained using antagomiRs and possibly other classes of pharmacological miRNA inhibitors should be interpreted with a certain degree of caution. Of note, similar discrepancies between pharmacological miRNA inhibition and genetic ablation have been described for another miRNA and other pharmacologic inhibitors [13], [25].

In summary, it appears that Treg do not express a specific miRNA essential for Treg function. Although absence of all miRNAs leads to spontaneous scurfy-like disease [4], [5], [6], no individual miRNA required for Treg function under homeostatic conditions has been described yet. The Treg-enriched miRNAs miR-155 and miR-10a are on their own largely dispensable for global Treg function under homeostatic conditions *in vivo* [26] and data not shown. Even the role of miR-146a remains unclear as the studies of miR-146a-deficient Tregs involved the co-transfer of miR-146a-deficient Treg along with miR-146a-deficient hyper-reactive IFN-γ secreting Teff and scurfy bone marrow as well as a pre-transplant preconditioning regimen [27]. Therefore, the function of miR-146a in Tregs under homeostatic conditions has not been determined. Thus, the absence of the protective layer of multiple miRNAs may predispose Treg to dysfunction, particularly in an inflammatory milieu and might constitute a risk factor for the development of autoimmune disease but absence of several individual miRNAs does not lead to complete Treg dysfunction.

## Materials and Methods

### RNA Isolation

Total RNA was extracted using Qiagen’s miRNeasy kit or Trizol (Invitrogen).

### miRNA Arrays

CD4^+^CD25^−^GFP^−^ conventional T cells (Tconv) and CD4^+^CD25^hi^GFP^+^ Treg from pooled axillary, cervical and inguinal lymph nodes from three pooled female FoxP3-GFP-hCre reporter mice were sorted on a Moflo flow cytometer [4]. Total RNA was extracted using Qiagen’s miRNeasy kit according to the manufacturer’s protocol; RNA integrity was tested using an Agilent Bioanalyzer Chip. 300 ng total RNA each was hybridized to Exiqon MirCury V9.2 arrays. Slides were scanned using GenePix Pro 6.0.0.29 on a GenePix 4000B scanner at 10 µm. All data is MIAME compliant and the raw data has been deposited in Gene Expression Omnibus (GEO), accession number GSE30847.

### miRNA RT-qPCR

RNA was isolated as described above. 20–30 ng total RNA were used for reverse transcription (RT) if not indicated otherwise. RT reactions were performed with ABI’s TaqMan MicroRNA RT kit (Cat No 4366596) according to the manufacturer except for using only 50% of the recommended volumes for the Applied Biosystems kits. PCR reactions were performed on 1 µl RT product with 50% of the recommended volumes for ABI reagents. As indicated, for some experiments primers and probes were designed as described [28] and ordered from Integrated DNA Technologies (IDT) using the following sequences: http://urology.ucsf.edu/blellochlab/protocols/miRNAqPCRsequences.txt.

Annealing temperatures for IDT reactions were: mmu-miR-10a, mmu-miR-16, mmu-miR-155, hsa-miR-155∶47.5°C. All reactions were normalized to sno202, measured with the ABI kit mouse snoRNA202 TaqMan assay, identification # 1232, (Cat No 4380914).

### Antagomirs

Antagomirs were designed as described [12]. Briefly, RNA molecules 100% complementary to the mature miRNA to be antagonized were synthesized by Integrated DNA Technologies (IDT www.idtdna.com) with the following rules: a) >19nt length b) 3′ Cholesterol moiety c) complete 2′-O-methylation d) phosphorothioate linkage: first two nt at 5′ end and 4 nt at 3′ end. Specific sequences are shown in Table S2. Lyophilized antagomiRs were reconstituted in H_2_O and directly added to the culture medium with at least dilutions of 1∶50.

### Mice

miR-10a-deficient mice were backcrossed for >7 generations to B6 (Gustavo Stadthagen & Anders Lund, unpublished results). C57BL/6J (stock Number 000664), NOD/ShiLtJ (Stock Number 001976), 129X1/SvJ (stock number 000691), DBA/2J (stock Number 000671) and BALB/cJ (stock number 000651) were purchased from the Jackson laboratories. 129S6/SvEvTac were purchased from Taconic. Foxp3-GFP-hCre and ROSA26-YFP mice have been described [4]. Mice were housed and bred under specific pathogen-free conditions at the University of California, San Francisco (UCSF) Animal Barrier Facility. Animal experiments were approved by the Institutional Animal Care and Use Committee (IACUC) of UCSF, approval numbers AN083988-01 and AN082188-02.

### Antibodies, Flow Cytometry and FACS Sorting

Flow cytometry and fluorescence activated cell sorting was performed as described [2], [4].

### iTreg Induction

After staining with mAb anti-CD4 PE-Cy7 (clone RM4-5) naïve CD62L^hi^CD4^+^GFP^-^ cells were sorted from pooled LN (axillary, cervical, inguinal, mesenteric, pancreatic and pelvic para-aortal) and spleen cells [8], activated with anti-CD3 (2C11)/anti-CD28 (PV-1) mAbs (0.5 µg/ml each) with 20 ng/ml human recombinant TGF-β (R&D). Retinoic acid (Sigma Aldrich R2625) diluted in DMSO (Sigma Aldrich) was added at 2.5 nM final concentration. After 4 or 5 days cells were harvested, washed, counted and stained with mAb anti-CD4 Alexa647 (clone GK1.5), then purified with a MoFlo cell sorter into GFP^−^ and GFP^+^ CD4^+^ T cells.

### Data Presentation

We present the data according to the editorial “How robust is your data?”, Nature Cell Biology, June 1, 2009. Figures show representative experiments but multiple individual experiments were provided as supporting online Figures to demonstrate reproducibility. Specifically, Fig. S1 shows individual experiments related to Figure 2a. Fig. S2 shows individual experiments related to Figure 2b,c and Fig. S3 shows individual experiments related to Figure 3.

## Supporting Information

Figure S1 **Reproducibility of the applied miRNA qPCR approach.** Shown are all 4 individual experiments for main Figure 2a, demonstrating the high reproducibility of the applied qPCR technology. Experiment 2 is shown in Figure 2a. All experiments were performed on different days using separate mice.
(EPS)

Figure S2 **Reproducibility of the applied miRNA qPCR approach.** A: The second experiment performed to generate Figure 2. B-E: Individual experiments performed to generate Figure 2b and 2c. B+C: The same experiment, LN and spleen analyzed separately. D: Different experiment than in B+C, done on a different day with a different mouse. This time LN and spleen cells were pooled. E: Different experiment than in B+C, done on the same day as D but with a different mouse. LN and spleen cells were pooled.
(EPS)

Figure S3 **Reproducibility of the applied miRNA qPCR approach.** Shown are both individual experiments performed for main Figure 3. Experiment 1 is shown in the main Figure 3 because the fold change is less extreme. Like Figures S1 and S2 this demonstrates the high reproducibility of the applied qPCR technology. We believe that for the conclusions drawn, the pattern of miRNA expression is more important than the fold change. Both experiments were performed on different days using different mice.
(EPS)

Table S1 **Tconv and Treg miRNA expression signature.** 300 ng total RNA was hybridized for Tconv and Treg to Exiqon MirCury V9.2 arrays. miRNA name (Column A), Fluorescence values of GFP- Tconv (Cy5) (Column B) and GFP+ Treg (Cy3) (column C). Fold fluorescence difference of Treg/Tconv (Column D). Background fluorescence, indicated as B635 and B532, was subtracted from total fluorescence for each spot on the slide.
(XLS)

Table S2 **AntagomiR sequences.** Nucleotide sequence and chemical modifications of antagomiR sequences used.
(XLS)
